# *Curcuma longa* Extract Exerts a Myorelaxant Effect on the Ileum and Colon in a Mouse Experimental Colitis Model, Independent of the Anti-Inflammatory Effect

**DOI:** 10.1371/journal.pone.0044650

**Published:** 2012-09-12

**Authors:** Rita Aldini, Roberta Budriesi, Giulia Roda, Matteo Micucci, Pierfranco Ioan, Antonia D’Errico-Grigioni, Alessandro Sartini, Elena Guidetti, Margherita Marocchi, Monica Cevenini, Francesca Rosini, Marco Montagnani, Alberto Chiarini, Giuseppe Mazzella

**Affiliations:** 1 Ospedale Policlinico S.Orsola and Dipartimento di Scienza dei Metalli, Elettrochimica e Tecniche Chimiche, Università degli Studi di Bologna, Bologna, Italy; 2 Dipartimento di Scienze Farmaceutiche, Università degli Studi di Bologna, Bologna, Italy; 3 Dipartimento di Medicina Clinica, Università degli Studi di Bologna, Ospedale Policlinico S. Orsola, Bologna, Italy; 4 Dipartimento di Medicina Interna, dell’Invecchiamento e Malattie Nefrologiche, Università degli Studi di Bologna; Ospedale Policlinico S. Orsola, Bologna, Italy; Concordia University Wisconsin, United States of America

## Abstract

**Background:**

Curcuma has long been used as an anti-inflammatory agent in inflammatory bowel disease. Since gastrointestinal motility is impaired in inflammatory states, the aim of this work was to evaluate if *Curcuma Longa* had any effect on intestinal motility.

**Methods:**

The biological activity of Curcuma extract was evaluated against Carbachol induced contraction in isolated mice intestine. Acute and chronic colitis were induced in Balb/c mice by Dextran Sulphate Sodium administration (5% and 2.5% respectively) and either Curcuma extract (200 mg/kg/day) or placebo was thereafter administered for 7 and 21 days respectively. Spontaneous contractions and the response to Carbachol and Atropine of ileum and colon were studied after colitis induction and Curcuma administration.

**Results:**

Curcuma extract reduced the spontaneous contractions in the ileum and colon; the maximal response to Carbachol was inhibited in a non-competitive and reversible manner. Similar results were obtained in ileum and colon from Curcuma fed mice. DSS administration decreased the motility, mainly in the colon and Curcuma almost restored both the spontaneous contractions and the response to Carbachol after 14 days assumption, compared to standard diet, but a prolonged assumption of Curcuma decreased the spontaneous and Carbachol-induced contractions.

**Conclusions:**

Curcuma extract has a direct and indirect myorelaxant effect on mouse ileum and colon, independent of the anti-inflammatory effect. The indirect effect is reversible and non-competitive with the cholinergic agent. These results suggest the use of curcuma extract as a spasmolytic agent.

## Introduction

Ulcerative colitis (UC) and Crohn’s disease (CD) are chronic inflammatory conditions of the intestinal tract: they are the consequence of environmental influences, genetic disorders, intestinal microbiota alterations, leading to an abnormal immune response with inflammation [1], [2]. A combination of all these factors is probably necessary for clinical expression of the disease [3]. Although the inflammation may remain limited to certain gastrointestinal segments, the function of the whole tract is altered [4], [5], [6]. Intestinal motility has been reported to be impaired in patients with Inflammatory Bowel Disease (IBD) [7], [8]. Inflammation is associated with decreased colonic mixing and haustra formation, but increased propulsive motility, leading to diarrhea [9]. The physiopathology of dysmotility in IBD has not been completely elucidated: a selective hyperactivity 10] of the non-adrenergic, non-cholinergic inhibition has been documented in human colon. Disruption in intracellular Ca2+ mobilization and signaling [11], defective norepinephrine release [12], impairment of the protease-activated receptor 2 [13], defective inhibitory neuron dependent relaxation in the colon [14] have been reported in the experimental animal. Conventional therapy for IBD is hampered by toxicity, intolerance or slow onset of action [15]. Recently, anti-tumour necrosis factor alpha and other biologic agents have been introduced [3], [16]–[18], but their safety in long term treatment has not been proved jet. Therefore, the use of natural substances with no side effects, though not so powerful effective as drugs so far employed, is encouraged, at least in the prevention and in the maintenance of remissions of IBD. Unfractionated or low-molecular-weight heparin, omega-3 polyunsaturated fatty acids, microbes and microbial products are currently under investigation [3].

*Curcuma longa* (turmeric) is a perennial herb, cultivated in the Southeast Asia [19]. In traditional medicine it has been used for centuries due to its antitumor, antimicrobial, anti-inflammatory, antioxidant properties and it presents acethylcholinesterase inhibitory activity [20]–[25].

Turmeric chemicals include curcumin (the primary constituent responsible for its yellow color), demethoxycurcumin, and bisdemethoxycurcumin, as well as volatile oils, sugars, proteins, and resins, curcumin (1,7-bis[4-hydroxy-3-methoxyphenyl]-1,6-heptadiene-3,5-dione) being the most studied compound [26]. The anti-inflammatory activity of curcumin has been investigated in various in vitro and in vivo studies [27]–[30]. In the experimental animal a turmeric extract has been shown to prevent the development of Trinitrobenzene Sulfonic Acid (TNBS)-induced colitis [28], [31]–[33] through the inhibition of signal transduction pathways critical to inflammatory responses, such as AP-1, protein kinase C, and NF-*κ*B [34], [35]–[37]. Moreover, it has been shown to prevent inflammation through blockage of NF-*κ*B in the mucosa in Dextran Sodium Sulphate (DSS) induced chronic colitis [38] and to inhibit immunostimulatory functions of dendritic cells by blocking MAPKs and NF-*κ*B activation [39]. In addition, Curcuma, in a randomized, double-blind, placebo-controlled trial, has been shown to be effective and safe in maintaining ulcerative colitis remission, and to decrease active oxygen species production [30]. So far, many data are available about *Curcuma longa* anti-inflammatory properties, but little and conflicting information is available about the effect of *Curcuma longa* on intestinal motility [40]–[42]. The wide clinical use of *Curcuma longa* as an anti-inflammatory agent in IBD and its empiric use in diarrhea in eastern countries prompted the present investigation in order to evaluate whether Curcuma exerts some myorelaxant effect on intestinal ileal and colonic motility in healthy intestinal segments, whether this effect, if any, is also observed in DSS induced acute and chronic experimental colitis, and, finally, whether this effect is independent of the anti-inflammatory effect.

**Figure 1:**
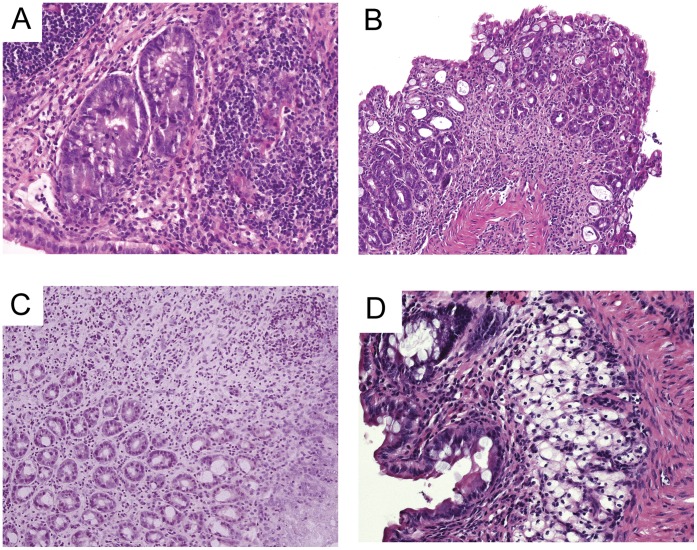
Photomicrographs of hematoxylin and eosin staining of Colon sections from DSS (5%w/v over 7 days) treated mice, before(a) and after 7 days Curcuma extract administration (b) and from DSS (2.5%,w/v, three cycles over 52 days) (c) and after 21 days Curcuma extract administration (d). (**a**) Intestinal mucosal with edema and marked/moderate acute inflammation composed by neutrophils, lymphocites and plasmacells. The intestinal glands show moderate regenerative hyperplasia.(H&E 10×). (**b**) Moderate mucosal inflammation composed by lymphocytes and some neutrophils; the intestinal glands show mild regenerative hyperplasia. (H&E 5×). (**c**) Chronic colitis. The mucosa shows a chronic infiltrate composed by lymphocytes and plasmacells. The glands show mild regenerative hyperplasia. (H&E 5×). (**d**) Intestinal mucosal with mild inflammation composed by lymphocytes, plasmacells and rare neutrophils. The submucosa shows many foam macrophages (H&E 20×).

## Methods

### Animals

One hundred thirty male Balb/c mice (8 weeks old, 25–30 g b.w.) (Charles Rivers Laboratories, Calco, LC, Italy) were enrolled: a higher number of animals than required were recruited in order to compensate for the drops out in the course of the experiments, due to the severity of colitis compelling to stop the experiments, according to the Recommendations of the Ethical Committee of the University of Bologna for Animal Experiments. The animals were kept at constant light/dark cycling and constant room temperature of 22°C. They were fed the usual commercial diet and were allowed water *ad libitum*.

**Table 1:** Histological score.

Grade	Morphological features
**0**	No inflammatory infiltration of neutrophyls and eosinophils in the lamina propria.
**1**	Mild and focal infiltrate of neutrophils and eosinophils in the lamina propria with mild crypt aggression.
**2**	Moderate infiltrate of neutrophils and eosinophils in the lamina propria with moderate crypt aggression.
**3**	Marked and diffuse infiltrate of neutrophils and eosinophils in the lamina propria with marked crypt aggression.

**Table 2:** Histological score of inflammatory damage of the colon in mice after acute and chronic DSS induced colitis and after curcuma extract (200 mg/kg/day) or standard diet administration: range of 6 animals.

		Histological Grading			
		After colitis induction	After 7 days	After 14 days	After 21 days
**Controls**		0	0	0	0
**Acute** **colitis**	**followed by standard diet**	3–3	2–3		
	**followed by curcuma extract**	3–3	1–2		
**Chronic colitis**	**followed by standard diet**	3–3	2–3	2–3	2–2
	**followed by curcuma extract**	2–3	2–2	1–2	0–1

The histological score was performed by blind pathology to the study design.

Mice were divided into 5 groups: a first group of healthy animals (n 15) was used as donors of intestinal segments in order to evaluate the in vitro effect of Curcuma extracton intestinal motility parameters by antagonism of Carbachol-induced contraction in isolated ileum and distal colon. In a second and a third group of animals Dextran Sodium Sulphate (DSS) (MP Biomedicals, Solon; OH, USA; m.w. 36.000–50.000) chronic (n 50) and acute(n 25) colitis (see below) respectively were induced and the effect of DSS on the Disease Activity parameters and intestinal motility were evaluated. After colitis induction, Curcuma extract was orally administered (200 mg/kg b.w./day) and, at fixed time intervals, (see below), the Disease Activity parameters and intestinal motility were evaluated. A fourth group of animals (n 14) was used as controls and a fifth group of mice (n 6) were used as Curcuma fed control, after oral administration of Curcuma over seven days. For all groups of animals, intestinal histology, spontaneously and Carbachol-induced motility were assessed. The effect of Atropine in Carbachol-induced contraction was also studied in order to get more insights into the mechanism of action of Curcuma extract on the ileal and colonic contraction.

**Figure 2:**
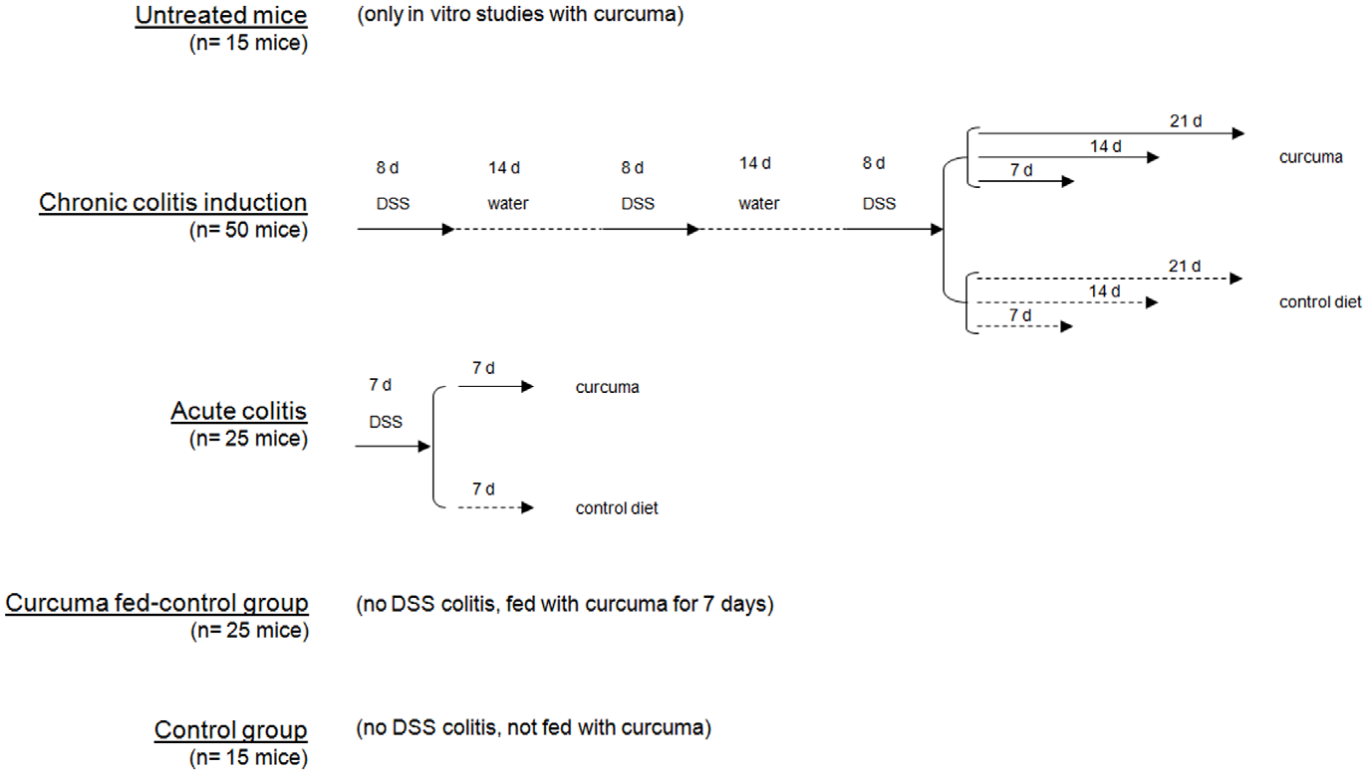
A schematic representation of the experimental design.

#### Study protocol

*In vitro studies*. The in vitro effect of curcuma was evaluated on isolated mice ileum and distal colon segments.

**Figure 3:**
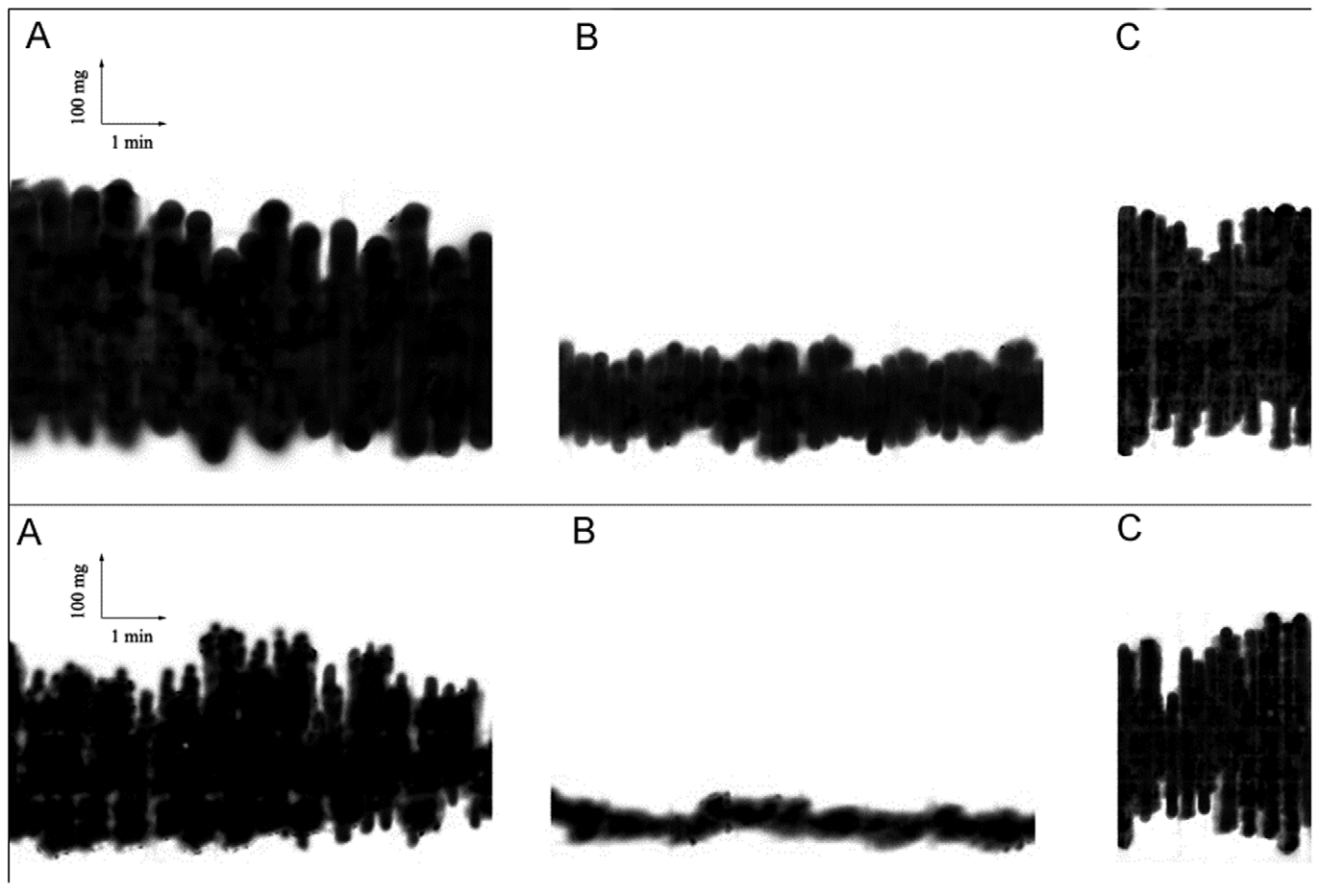
Inhibition of spontaneous motility of ileum (upper panel) and colon (lower panel) by curcuma extract. The segments were suspended in organ baths containing gassed warm Krebs solution under a load of 1 g maintained at 37°C. Tension changes in longitudinal muscle length were recorded. In control conditions (a), after curcuma extract additioned medium (b) and after washing (c).For details see Methods section.

#### In vivo studies

Chronic colitis: chronic colitis was induced in mice by oral administration of DSS (2.5%, w/v) in drinking water (see Text S1). Throughout the period, the animals were fed the usual commercial diet. After 52 days colitis induction, mice were divided into two groups: one group was switched to a Curcuma (200 mg/Kg b.w.) (Indena, Milan, Italy) added diet, the other continued the usual diet. Throughout the experiment, the Disease Activity Index (DAI) was evaluated. After colitis induction and after 7, 14, 21 days of either Curcuma extract added diet (200 mg/kg/b.w.) or standard diet administration, 6 mice of each group were sacrificed and the following parameters were evaluated: colon length, intestinal histology and motility. Acute colitis: acute colitis was induced in mice, by addition of DSS (5% w/v) in the drinking water for 7 days. After this period, the mice were randomly allotted to two groups, one group continued the standard usual diet, the other was switched to a diet similar to the previous but added with Curcuma (200 mg/kg b.w.); after 7 days, the animals were sacrificed. The same parameters as in the previous group were recorded. **Control animals.** 14 mice were studied as controls and the same parameters as above reported were evaluated.

**Figure 4:**
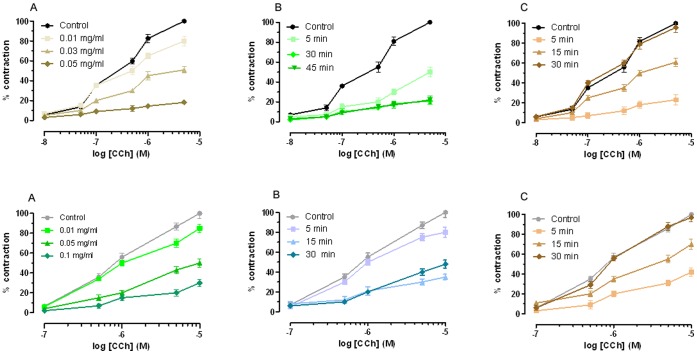
Effect of curcuma extract on carbachol-induced contraction in isolated mouse ileum (A) and distal colon (B). **a)** Cumulative concentration-response curves were obtained before and after exposure to curcuma extract for 30 min. Each point is the mean ± SEM (*n*  = 3–5). **b)** Time course of curcuma extract effect on Carbachol-induced contraction in isolated mouse ileum (**A**) or distal colon (**B**) (100%). Cumulative concentration-response curves were obtained before and after exposure to curcuma extract (0.05 mg/mL) for 5, 30 and 45 min. Each point is the mean ± SEM (*n*  = 3–5). **c)** Time course of curcuma extract (0.05 mg/mL) on carbachol-induced contraction in isolated mouse ileum (**A**) and distal colon (**B**). Cumulative concentration-response curves were obtained before and after exposure to curcuma extract (0.05 mg/mL) and following 5, 30 and 60 min washing. Data are the mean ± SEM (*n*  = 3–5). Where error bars are not shown, these are covered with the point itself.

#### Curcuma fed controls

6 mice were fed Curcuma added diet over 7 days and thereafter sacrified and similarly studied. A schematic representation of the study is reported in Figure 1.

### DSS Colitis Induction

Acute and chronic colitis were induced by DSS administration according to Wirtz [43] (see Text S1). The chronic colitis model has been shown to induce a long lasting colonic inflammation in mice [43].

**Figure 5:**
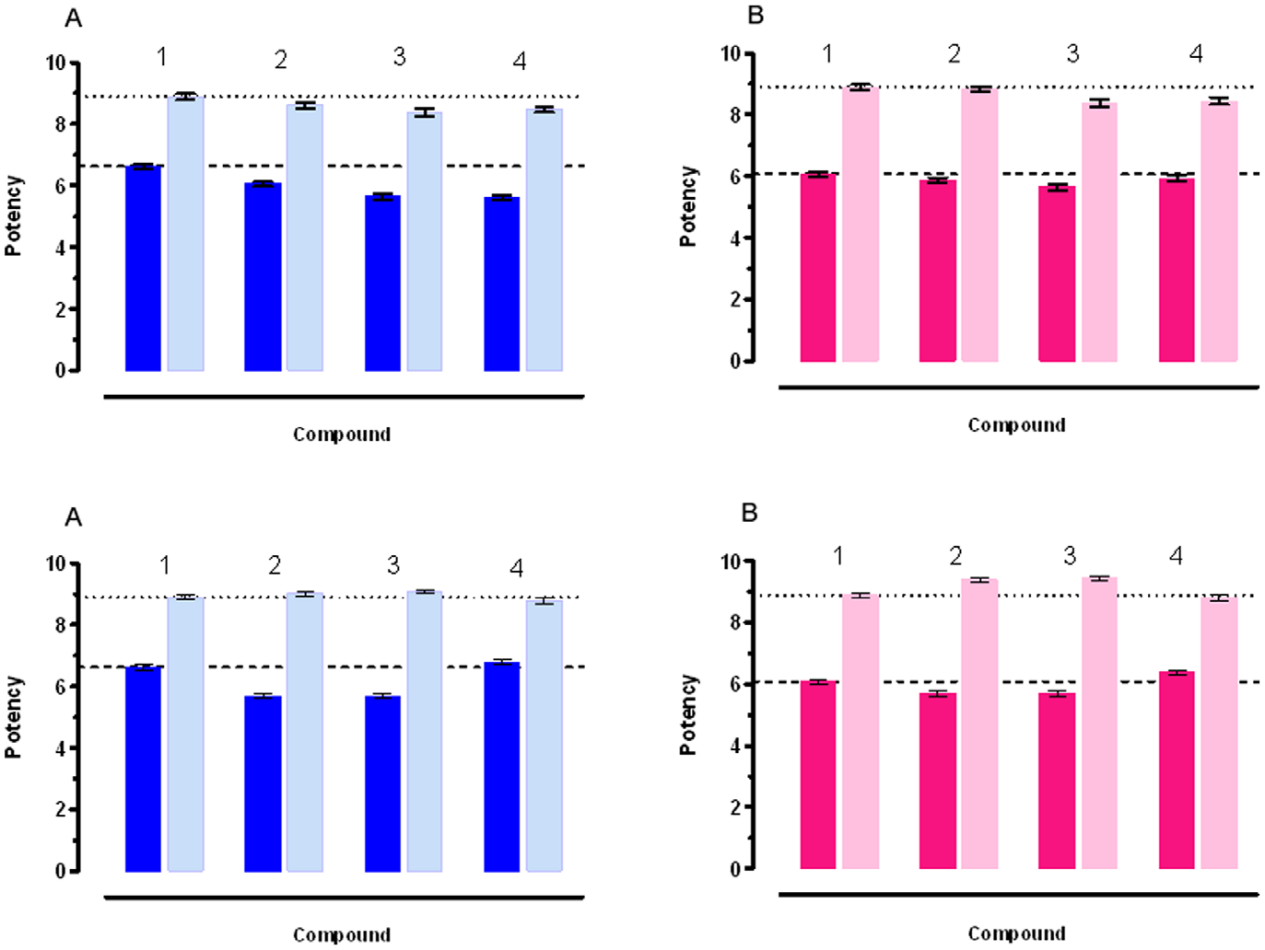
Potency of CCh (pEC_50_) (dark blu) and atropine (pA_2_) (light blu) on ileum (a) and on distal colon (red) and (pink) respectively. (1): control mice. (2): mice with acute DSS induced colitis; (3): acute colitis mice fed the usual diet over 7 days after stopping DSS; (4): acute colitis mice fed curcuma over 7 days after stopping DSS. The dashed line shows the control values for CCh pEC_50_. The dotted line shows the control values for Atropine pA_2_. When error bar are not shown these are covered by the point itself. The contraction induced by Carbachol and the antagonistic effects of Atropine were used as reference. In healthy animals Carbachol is by 3.55 times more potent on the ileum than on the colon. Atropine presents similar antagonistic activity on both tissues: these data have been used as a comparison with the effect of Carbachol and Atropine on tissues from acute colitis animals. Carbachol pEC_50_ decreased by 3.80 times in the ileum and 1.58 times in the colon and Atropine p*A*_2_ was reduced by 1.86 and 1.12 times respectively in the ileum and the colon, compared with the corresponding intestinal segments of control mice. Curcuma administration improved the response to Carbachol in the colon, but not in the ileum, also if it did not completely restore the normal values.

### Assessment of Colitis

#### Evaluation of disease activity index (DAI )and colon length

Disease Activity Index (DAI) was calculated for each animal as described by Fitzpatrick et al. [44]. The colon was individuated as the segment between the ileo-cecal valve and the rectum and the length was measured (see Text S1 and Table S1).

**Table 3:** Agonist (carbachol) and antagonist (atropine) affinities expressed as pEC_50_ or p*A*_2_ respectively on the isolated mice ileum and distal colon of control mice and mice fed Curcuma extract over seven days.

			Standard Diet^a^	CurcumaExt^b^
**ileum**	**CCh**	**pEC_50_** c	6.63±0.04	6.40±0.03
	**Atropine**	**p** ***A*** **_2_** d	8.89±0.03	9.16±0.02
**colon**	**CCh**	**pEC_50_** c	6.08±0.01	6.00±0.01
	**Atropine**	**p** ***A*** **_2_** d	8.89±0.01	9.47±0.02

a Tissues from healthy mice fed standard diet over seven days.

b tissues from healthy mice fed Curcuma extract over seven days.

c pEC_50_ =  –log EC_50_. EC_50_ values are the means ± SE of at least four independent experiments and were calculated by a non linear regression curve-fitting computer program (47).

d p*A*_2_ values ± SE were calculated from Schild plots [48] constrained to slope –1.0 [49]). p*A*_2_ is the positive value of the intercept of the line derived by plotting log (DR –1) vs log [antagonist]. The log (DR –1) was calculated from three different antagonist concentrations, and each concentration was tested from four to six times. Dose-ratio (DR) values represent the ratio of the potency of the agonist carbachol (EC_50_) in the presence of the antagonist and in its absence. Parallelism of concentration–response curves was checked by linear regression, and slopes were tested for significance (p<0.05).

### Curcuma Extract Administration

Mice were on a 4RF21diet (Mucedola S.r.l., Milan, Italy).The 4RF21 complete food was added with Curcuma (Indena Spa, Milan, Italy) extract at a final concentration of 1 g/kg. The delivery form of Curcuma used in the present study is a patented formulation of Curcumin (Free Curcumin; Curcuminglucuronide; Curcuminsulphate), a dietary phenolic, with soylecithin. The two compounds form a non-covalent adduct in a 1∶2 weight ratio, and two parts of microcrystalline cellulose are then added to improve formulation (www.phytosomes.info).

**Figure 6:**
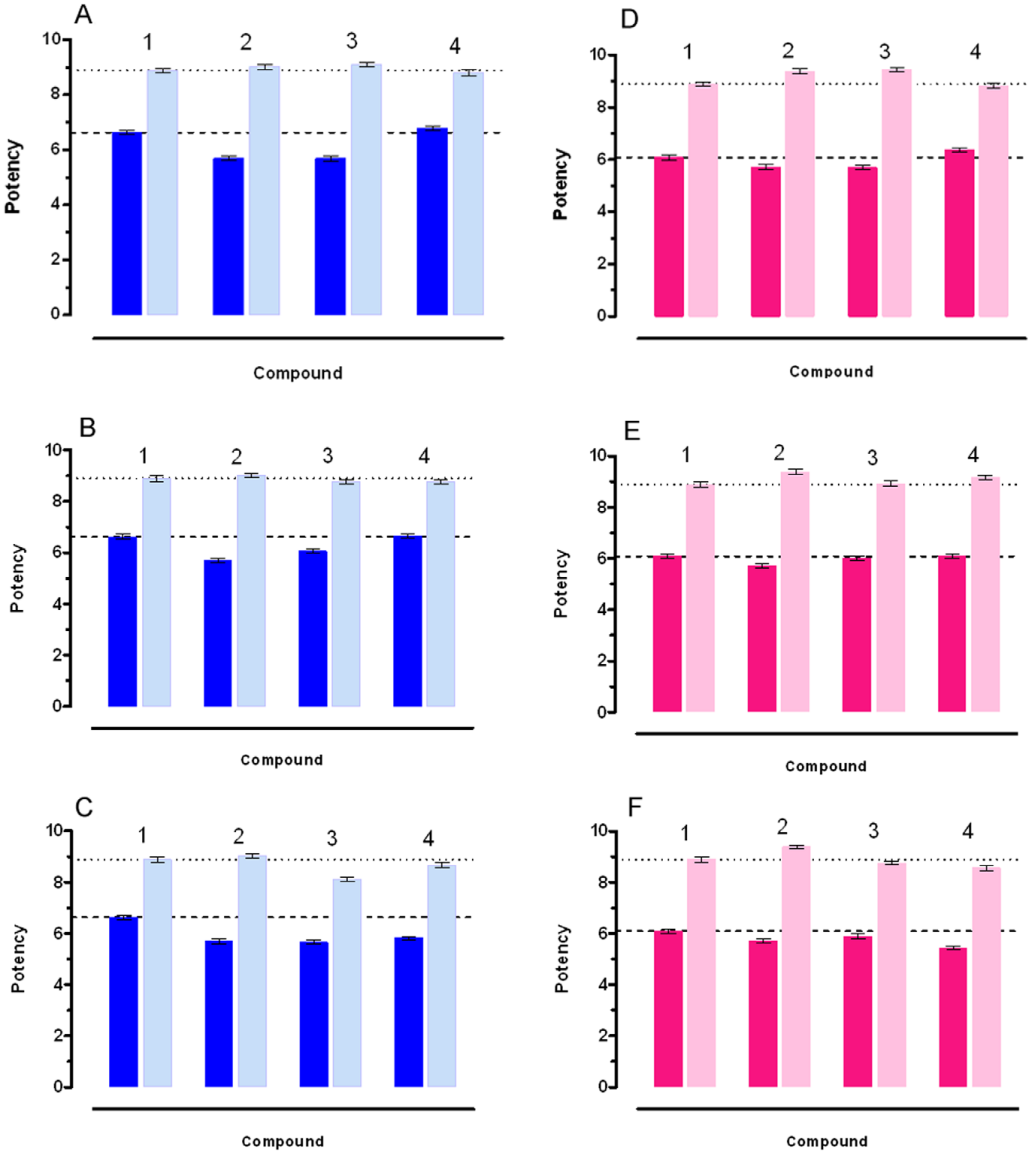
Potency of CCh (pD_2_) and Atropine (pA_2_) on the ileum (a, b, c) and on the colon (d, e, f) from mice with chronic colitis. 1: control mice, 2: chronic DSS induced colitis, 3: DSS chronic colitis mice assuming standard diet over 7 (a) 14 (b) and 21 (c) days after stopping DSS; 4: DSS chronic colitis mice assuming curcuma extract over 7 (a), 14 (b) and 21 (c) days after stopping DSS. The dashed line shows the control values for CCh pEC_50_. The dotted line shows the control values for Atropine pA_2_. When error bar are not shown these are covered with the point itself Carbachol. The contraction induced by Carbachol and the antagonistic effects of Atropine were used as reference. In healthy animals Carbachol is by 3.55 times more potent on the ileum than on the colon. Atropine presents similar antagonistic activity on both tissues: these data have been used as a comparison with the effect of Carbachol and Atropine on tissues from chronic colitis animals. After induction of chronic colitis, Charbacol pEC_50_ decreased by 8.71 times in the ileum and 2.34 times in the colon and Atropine p*A*_2_ was increased by 1.35 and 3.09 times in the ileum and the colon respectively, compared with the corresponding intestinal segments of control mice. Curcuma administration improved the response to Carbachol in the ileum by 9.12 times and by 2.45 times in the colon after 14 days administration; Atropine pA_2_ was decreased by 1.78 and 1.66 respectively in the two intestinal segments.

The chemical preparation was proved to be 18–22% pure by HPLC total curcuminoids content. Each mouse received 200 mg/kg per day, i.e. the human equivalent dose for mice [45] (see Text S1).

#### Ethics statement

The work has been conducted according to relevant National and International Guidelines. All experiments were conducted in conformity with the Public Health Service Policy on Humane Care and use of Laboratory Animals and approved by the Ethical Committee of the University of Bologna(PR 22.03.10). The animals were kept at constant temperature, light/dark cycle. Whenever a mouse gave signs of discomfort, the experiment was interrupted and the animal was switched to plain water and the usual commercial diet and excluded from the study.

### Experimental Procedure

Twenty four hours before the experiments, food was withdrawn and water was maintained *ad libitum*. The animals were sacrificed by cervical dislocation. The abdomen was opened by median laparotomy and exposed. A portion of jejunum, immediately after the Treitz was removed (1.5 cm) and retained for histological examination. A 1.5 cm length segment of the terminal ileum, immediately proximal to the ileo-cecal valve and a 2.5 cm region of the distal colon were identified, slightly cleaned with Krebs solution to remove fecal residues and dissected into two parts: one part was placed in 10% formalin for subsequent histological analysis, a second was retained for immediate in vitro motility studies (see below). Stools were analyzed for consistency and blood traces.

### Histology

The sections of ileum and colon specimens were preserved in 10% neutral buffered formalin for about 48 hours and routinely processed. Five micron sections were cut and stained with Haematoxylin and Eosin (H&E).The histological preparations were examined histologically on a blinded basis.

#### Histological grading

Acute colitis is characterised by intense inflammatory cell infiltrate, crypt abscesses, mucin depletion, and surface ulceration, that are the main histological features of acute activity. The hallmark is the presence of neutrophils and eosinophils infiltrating lamina propria and crypt epithelium.

Chronic colitis is characterized chronic inflammatory cells, plasma cells and lymphocytes in the lamina propria, distortion of the normal architecture of colonic mucosa, including crypts branching and regeneration. These morphological features induce a variety of altered shapes and sizes of crypts and changes in surface contours. Basal accumulation of lymphocytes and plasma cells with hyperplasia of lymphoid tissue probably represents an early immunologic manifestation of the underlying disease.

Therefore, according to the amount of acute and chronic inflammatory cell infiltrate in colon and in small bowel slides colitis was classified in four grades as reported in Table 1.

### Functional Studies

The animals were sacrificed by cervical dislocation, and the organ (ileum and distal colon) required was set up rapidly under a suitable resting tension in 15 mL organ bath containing appropriate physiological salt solution (PSS) consistent warmed (see below) and buffered to pH 7.4 by saturation with 95% O_2_–5% CO_2_ gas.

#### Mouse ileum

The terminal portion of ileum (immediately proximal to the ileo-caecal junction) was cleaned, and segments 1 cm long of ileum were set up under 1 g tension at 37°C in organ baths containing Tyrode solution of the following composition (mM): NaCl, 145; KCl, 2.6; CaCl_2_·2H_2_O, 1.5; MgCl_2_·6H_2_O, 0.73; NaH_2_PO_4_·2H_2_O, 0.33; NaHCO_3,_ 4.8; glucose, 11.1 [46]. Each segments was set up under 1 g tension in the longitudinal direction along the intestinal wall. Tissue were allowed to equilibrate for at list 30 min during which time the bathing solution was changed every 10 min. Concentration-response curves were constructed by cumulative addition of the agonist Carbachol (CCh). The concentration of agonist in the organ bath was added only after the response to the previous addition had attained a maximal level and remained steady. Contractions were recorded by means of displacement transducer (FT. 03, Grass Instruments, Quincy, MA) using Power Lab software (ADInstruments Pty Ltd, Castle Hill, Australia). In all cases, parallel experiments in which tissues did not receive any antagonist were run in order to check any variation in sensitivity. Concentration–response curves to agonist were obtained at 30 min intervals, the first one being discarded and the second one used as control. A new concentration-response curve to agonist was obtained following incubation with the antagonist (Atropine or Curcuma extract). Tension changes were recorded isotonically.

#### Mouse distal colon

A segment of about 2 cm of the distal colon was transected, rinsed with Krebs solution of the following composition (mM): NaCl, 119; KCl, 4.5; CaCl_2_, 2.5; MgSO_4_·7H_2_O, 2.5; KH_2_PO_4_·2H_2_O, 1.2; NaHCO_3,_ 25; glucose, 11.1; the mesenteric tissue was removed. The segments were suspended in organ baths containing gassed warm Krebs solution under a load of 1 g maintained at 37°C. Tension changes in longitudinal muscle length were recorded. Tissues were allowed to equilibrate for at least 30 min during which time the bathing solution was changed every 10 min. Concentration–response curves to agonist Carbachol (CCh) were recorded isotonically and obtained at 30 min intervals, the first one being discarded and the second one taken as control. Following incubation with the antagonists (Atropine or Curcuma extract), a new concentration–response curve to agonist was obtained. Longitudinal muscle contractions were recorded isotonically by the mean of force displacement transducer (FT 03, Grass Instruments, Quincy, MA) using Power Lab software (ADInstruments Pty Ltd, Castle Hill, Australia). In all cases, parallel experiments in which tissues did not receive any antagonist were run in order to check any variation in sensitivity.

### Statistical Analysis

#### Determination of Dissociation Constants

Functional activity of CCh and antagonism of Atropine and Curcuma extract *vs* CCh induced contraction, was determined in gut segments. The biological results of agonist CCh was expressed as pEC_50_ values. pEC_50_ values represent the –logEC_50_. EC_50_ values are the means ± SE of at least four independent experiments and were calculated by a non-linear regression curve-fitting computer program [47].

In functional experiments, dose ratios at the EC_50_ values of the agonist were calculated at three to five antagonist concentrations, and each concentration was tested from two to four times. The results are expressed as p*A*_2_ values [48], [49]. Data are presented as means ± SE of *n* experiments. Differences between mean values were tested for significance by Student’s *t*-test. *P* value less than 0.05 was considered significant.

The antagonism activity to CCh of curcuma extract was estimated by determining the concentration of the non-competitive antagonist that inhibited 50% of the maximum response to the agonist. Three different antagonist concentrations were used and each concentration was tested at least three times. A pharmacological computer program was used to analyze data [49]. It was always verified that EC_50_ values for the agonist in tissues receiving only the solvent were not significantly different (*P*>0.05) from control values. In other cases experiments were discarded. Figures were created using GraphPad software [50].

### Materials

Carbachol, Atropine and all chemicals were obtained from Sigma (St. Louis, MO, USA). Curcuma was gently supplied by Indena (Indena Spa, Milan, Italy).

## Results

### Induction of Acute and Chronic colitis Assessment of Acute and Chronic Colitis and Evaluation of the Response to Curcuma Extract (see Text S1)

#### Histological parameters

the histological score is reported in Table 2. Both the ileum and the distal colon of the control mice demonstrated a very mild lymphocytic infiltration, typical for the “physiological inflammation” observed in murine intestine under normal conditions [51].

Acute DSS-induced colitis is characterized by histological findings such as severe edema, infiltration of inflammatory cells with granulocytes into both the mucosa and the submucosa, with destruction of the epithelial cells: seven days after withdrawal of DSS, the histological analysis indicated that the severity of colitis was higher in DSS-treated mice assuming standard diet compared to Curcuma treated mice. In chronic DSS induced colitis, chronic inflammatory cells sometimes with mild neutrophil infiltrate were present in the mucosa and submucos; in some cases we observed a mild epithelial aggression. Twenty days after suspension of DSS, Curcuma extract administration improved the histological findings compared to the standard diet control group (Figure 2). Not any pathological alterations were found in the duodenum and jejunum.

#### Colon length and Disease Activity Index (DAI)

the results are reported respectively in Figure S1, S2 and S3.

### Functional Studies

#### Spontaneous motility in ileum and colon

In control mice, spontaneous contractions in the ileum and colon were constant in amplitude and frequency. The ileal and colonic contractions in basal conditions were considered as 100% and the contractions in pathological conditions were compared with the control motility. In vitro curcuma extract inhibited the spontaneous intestinal contraction by 100% both in ileum and colon (Figure 3). In vivo, when orally administered over one week to healthy mice, the spontaneous motility in the ileum and colon was reduced by 30%; the contractions were constant in frequency and amplitude. In acute colitis, intestinal motility was increased by 230% in the ileum; the contractions were highly irregular in amplitude and frequency; they were reduced by 100% in the colon; Curcuma extract administration over one week to mice reduced the by 50% the spontaneous contraction in ileum without any significant effect on the colon, while standard diet administration did not modify significantly ileal and colonic contractions. In chronic DSS colitis, spontaneous contractions increased by 400%and 300% respectively in the ileum and colon. They were of irregular amplitude and frequency. Seven days after curcuma administration, a partial normalization was observed in both the ileum and the colon; 14 days after Curcuma administration the basal ileal and colonic motility were normalized and were of constant amplitude and frequency. A longer administration (21 days) inhibited the basal spontaneous activity of the ileum and colon. Standard diet administration did not substantially modify the intestinal basal motility since only a partial recovery was observed with time.

#### Response to Carbachol and Atropine

*Curcuma extract effect on isolated ileum and colon.* Initially we have evaluated the antispasmodic activity of *Curcuma longa Linn* Extract from rhizomes on ileum and distal colon. Experiments performed in mice ileum and colon showed that Curcuma extract inhibited the maximum response to Carbachol in a non-competitive manner. These results show that the extract produce a smooth muscle relaxation effect on mice ileum and colon, as previously described for Guinea Pig [42]. Furthermore, this blockade was reversed after 30 min tissue washing (Figure 3). The IC_50_ value for Curcuma extract was 0.03 mg/ml in ileum and 0.04 mg/min distal colon respectively (Table S2). In both cases, the agonist activity was comparable.

*Effect of Carbachol and Atropine on ileum and colon from control mice*. In order to evaluate the effects of Curcuma extract on the intestinal muscular layers of ileum and colon, we have used as reference the contraction induced by Carbachol and the antagonistic effects of Atropine. Figure 4 reports Carbachol pEC_50_ and Atropine p*A*_2_ on ileum and colon respectively.

As it can be observed (for data see Table S3), Carbachol is by 3.55 times more potent on the ileum than on the colon. Atropine presents similar antagonistic activity on both tissues: these data have been used as a comparison with the effect of Carbachol and Atropine on tissues from DSS treated animals (acute and chronic colitis ) and after Curcuma extract or placebo administration.

### Functional Study in DSS Mice Smooth Muscle

#### Acute colitis

Acute colitis was induced with DSS (5%, w/v) over 7 days in the drinking water, after which one group went on assuming the usual commercial diet, the other was switched to curcuma (200 mg/kg/day) added diet for 7 days.

Carbachol induced contraction was reduced: in fact Charbacol pEC_50_ decreased by 3.80 times in the ileum and 1.58 times in the colon compared to the respective control intestinal segments (Figure 5; for data see Table S4). Similarly, the effect of Atropine was decreased: p*A*_2_ was reduced by 1.86 and 1.12 times respectively in the ileum and the colon, compared with the corresponding intestinal segments of control mice. Both the effects were stronger in the ileum than in the colon: This finding is at variance with the histological damage, which is limited to the colon, resembling the histopathological features of UC. Seven days Curcuma extract administration improved the response to Carbachol in the colon, but not in the ileum, both in comparison with the response of colon from mice with acute disease and mice assuming control diet, also if it did not completely restore the normal values. The response to Atropine was decreased both in the Curcuma added diet group and in the controls. In the ileum, Curcuma extract administration did not even partially restore the response to Carbachol and Atropine.

In order to evaluate whether Curcuma extract had any effect on normal intestine and compare it, if any, with the effect of Curcuma on the intestine from mice with DSS induced acute colitis, we have administered Curcuma extract over 7 days to control mice. As shown in Table 3, Curcuma extract, when added to diet decreased the response to Carbachol in the ileum, by 1.69 and in the colon, by 1.20, while it increased in both the response to Atropine. As it can be observed, the effect was greater in the ileum.

#### Chronic colitis

Chronic colitis was induced in mice with DSS (2.5%w/v), three “8 days” cycles, with plain water between, for a total of 52 days, after which one group continued the control diet for 7 days, the other was switched to curcuma (200 mg/kg/day) over 7 days. After colitis induction, animals were either switched to curcuma extract administration or continued the usual diet. They were then sacrificed after 7, 14, 21 days and the two groups of animals were compared (Figure 6; for data see Table S5).

## Discussion

Scanty data are available in literature about the effect of Curcuma on intestinal motility. In healthy humans, an indirect evidence that Curcuma activates bowel motility has been produced by hydrogen breath [40]. On the contrary, Curcuma increases intestinal transit time in albino rats and ileal motility in Guinea Pig, consistently with the traditional use of curcumin in disorders of altered intestinal motility [41]–[42]. The use of turmeric as an anti-inflammatory agent in IBD and the traditional empiric use of Curcuma as an antidiarrheal symptomatic drug in functional disorders of the gastrointestinal tract suggested an effect on intestinal motility.

The GI tract function is under neural control, extrinsic autonomic nerves influencing the activity of enteric neurons located within the wall of the gut [52], [53]. Although the inflammation in IBD occurs in discrete regions of the gastrointestinal tract, the ensuing functional alterations, among which altered motility, are not limited to the inflamed segments [54], [55] and a reduced sympathetic regulation in uninflamed intestinal tracts has been demonstrated [12]. The development of intestinal dysmotility may in turn result in abnormal growth of intestinal microflora, subsequently inducing a translocation of bacteria or bacterial products through the impaired mucosal barrier [56].

In humans, it is under debate whether IBD is associated with hyper or hypomotility [7], [8], [57]. Reduced haustra formation, but increased colonic propulsive motility and diarrhea seem to be present [58], [59]. DSS colitis shares clinical and histopathological features of UC and reproduces a model of chemically driven colitis, where the damage of the epithelium is related to inflammation without an immune component. In our system, this model represents a good way to induce inflammation in the colon and to study its effect on motility. Conflicting data exist about the effect of DSS on intestinal motility in the experimental animal. Spontaneous phasic contractions and Carbachol-evoked contractions of distal colon smooth muscles have been found diminished by DSS [11], [60]–[62]. Conversely, increased Carbachol-evoked contractions and colonic smooth muscle hypercontractility were reported [63]. These findings may correlate with differences in experimental conditions, strain and gender differences in the susceptibility of DSS-colitis. In view of the possible gene differences, we have used male Balb/c mice: Balb/c mice are a strain commonly used for all-purpose laboratory investigations. Moreover males have been preferred over females due to the phasic influences of sexual hormones on gastrointestinal motility. In the present investigation, DSS induces deep disturbances in basal motor activity of ileum and colon: it is worthwhile noting that in acute colitis a hyperexcitability of the ileum is observed associated with a depressed motor activity in the colon, while a severe hyperexcitability is present both in ileum and in colon in the chronic colitis model. The abolition of any motor activity of the colon in acute experimental colitis is in agreement with the clinical condition of the toxic megacolon. We have chosen as reference of disturbed motility the ileal and colonic response to Carbachol and the inhibitory effect of Atropine on the Carbachol induced contraction. Carbachol and Atropine are respective agonist and antagonist at cholinergic muscarinic receptors. Both agonist and antagonist bind to the cholinergic receptors M(2) and M(3), present in mice small intestine and colon cellular membrane. In the present investigation, we have induced both chronic and acute colitis in mice, since the chronic model is more suitable for Curcuma treatment. The muscarinic receptor agonist Carbachol generated a smaller contractile force in the DSS-treated mice than in normal mice: this was the case both in the inflamed colonic strips and in the normal ileal segments. The altered response to the antagonist is a further confirmation that the cellular membrane, where not anatomically altered, as is the case with the ileum, is functionally altered. In this study we show that, in control conditions, Carbachol effect is more potent in the ileum than in the colon, while the antagonistic effect of Atropine is similar. In vitro, Curcuma exerts an inhibition of intestinal basal and Carbachol induced contraction both in the ileum and the colon: this effect is non-competitive and reversible. The inhibition of basal intestinal motility demonstrates a direct effect on the intestinal muscular cells. The inhibition of Carbachol response is stronger in the ileum while the antagonistic effect of Atropine is increased in both. When orally administered to healthy animals, Curcuma has similarly shown a direct myorelaxant activity and an inhibition of Carbachol response on the ileum and colon, stronger on the former than on the latter. The effect of Atropine is conversely increased on both the intestinal tracts. To our knowledge, this is the first report of a myorelaxant effect of *Curcuma longa* both in the normal ileum and colon and in an experimental colitis model. The present experiments, in agreement with most of the authors, confirm that Carbachol-induced contractions are reduced in the colon in the DSS-mouse colitis model: in addition, in the present investigation, defective Carbachol evoked contractions have been documented also in the ileum. In acute and chronic colitis, Curcuma extract added diet improves the ileal and colonic motility: in the chronic colitis model, 14 days Curcuma administration restores the motility pattern in the colon and in the ileum, but after a longer Curcuma extract oral administration, once the mucosal repair is achieved, the myorelaxant effect of Curcuma is prevalent in both the intestinal segments and the inhibitory effect of Curcuma on the colonic muscular layers is predominant, showing that the inhibition on the muscular layers is independent of the anti-inflammatory effect. The potency of the inhibitory effect of Curcuma Longa on the intestinal motility is not powerful: this is not surprising since Curcuma is a natural substance and this should be considered in the translation from the animal model to human pathology.

In conclusion, Curcuma extract exerts a myorelaxant effect on the ileum and colon independent of the anti-inflammatory effect. The mechanism of action is due both to a direct myorelaxant effect on the intestinal muscle layers and to a non-competitive and reversible inhibition of the cholinergic agent. It was beyond the aim of the present paper to investigate the mechanism underlying this effect. The present study provides the rationale for the use of Curcuma Longa in motility disorders of IBD and suggests a possible application to functional motor disturbances of the gastrointestinal tract, due to the myorelaxant effect on the normal intestine, independent of the anti-inflammatory activity.

## Supporting Information

Figure S1 **Effect of acute (a) and chronic (b) DSS administration on colon length, followed by curcuma extract (200 mg/kg) or standard diet.** In the acute colitis model mice received DSS (5%) in drinking water over 7 days followed by seven days water; in the chronic colitis model, they received DSS (2.5%) over 7 days followed by 14 days water per cycle, for a total of three cycles. After colitis induction they were fed either curcuma extract or standard diet respectively over 7 and 21 days. a1) control. a2) acute colitis.a3) acute colitis followed.by curcuma over 7 days. a4) acute colitis followed by standard diet over 7 days. b1) control. b2) chronic colitis. b3) chronic colitis followed by curcuma extract over 21 days. b4) chronic colitis followed by standard diet over 21 days. Each point is the mean ± SD (6 determinations). Significance: (**a**) 1*vs* 2: p<0.005; 1*vs* 3: p<0.005; 1*vs* 4: p<0.001; 2 *vs* 3: p<0.05; 2 *vs* 4: p<0.05; 3*vs* 4: p ns. (**b**) 1*vs* 2: p<0.0001; 1*vs* 3: p<0.005; 1*vs* 4: p<0.0005; 2 *vs* 3: p<0.01; 2 *vs* 4: p<0.05; 3 *vs* 4: p ns.
(TIF)

Figure S2 **The Disease Activity Index in the acute (a) and chronic colitis (b) model.** The DAIs were calculated as described in the Materials and Method section. Each point is the mean ± SD (6 determinations). Where error bars are not shown, these are covered by the point itself. Significance: (**a**) From day 4 on: DSS *vs* control tap water: p<0.0001. (**b**) From day 6 on: DSS *vs* control tap water: p<0.0001.
(TIF)

Figure S3 **The DAI in mice fed curcuma (red) or standard diet (black) after acute (a) and chronic (b) colitis induction.** The DAIs were calculated as described in the Materials and Method section. Each point is the mean ± SD (6 determinations). Significance: (**a**) 7 days after stopping DSS, Curcuma extract *vs* control diet, p<0.0001. (**b**) 14 days after stopping DSS, Curcuma extract *vs* standard diet: p<0.05; 21 days: p<0.005.
(TIF)

Table S1 **Parameters investigated for the evaluation of the Disease Activity Index.**
(DOC)

Table S2 **Antagonist affinities, expressed as IC_50_ Values, in the different mouse gut smooth muscle segments.**
(DOC)

Table S3 **Potency of Chrabacol and Atropine on isolated mice tissues.**
(DOC)

Table S4 **Agonist (Carbachol) and antagonist (Atropine) affinities expressed as pEC_50_ or p** ***A*** **_2_ respectively in the isolated mice ileum and distal colon.**
(DOC)

Table S5 **Agonist (Carbachol) and antagonist (Atropine) affinities expressed as pEC_50_ or p** ***A*** **_2_ respectively in the isolated mice ileum and distal colon.**
(DOC)

Text S1 **Supporting information.**
(DOCX)
