# Supplementary figures and images for: Curcuma longa Extract Exerts a Myorelaxant Effect on the Ileum and Colon in a Mouse Experimental Colitis Model, Independent of the Anti-Inflammatory Effect
Source: PLoS One. 2012 Sep 12;7(9):e44650. doi: 10.1371/journal.pone.0044650 (PMC3440350; doi:10.1371/journal.pone.0044650)

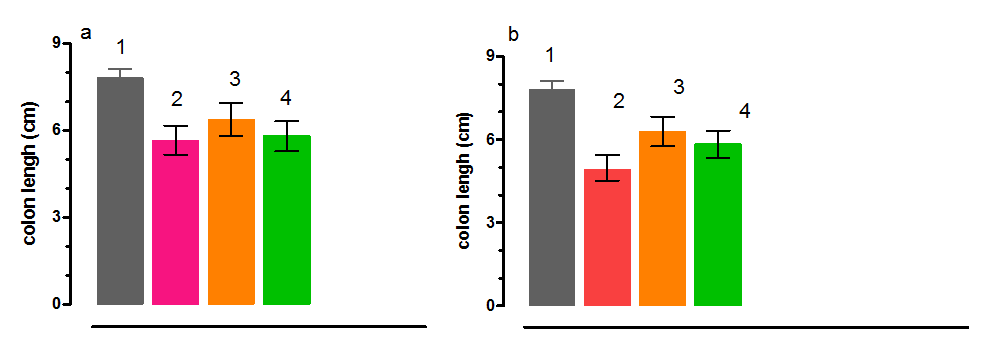

Supplement: Figure S1 — Effect of acute (a) and chronic (b) DSS administration on colon length, followed by curcuma extract (200 mg/kg) or standard diet. In the acute colitis model mice received DSS (5%) in drinking water over 7 days followed by seven days water; in the chronic colitis model, they received DSS (2.5%) over 7 days followed by 14 days water per cycle, for a total of three cycles. After colitis induction they were fed either curcuma extract or standard diet respectively over 7 and 21 days. a1) control. a2) acute colitis.a3) acute colitis followed.by curcuma over 7 days. a4) acute colitis followed by standard diet over 7 days. b1) control. b2) chronic colitis. b3) chronic colitis followed by curcuma extract over 21 days. b4) chronic colitis followed by standard diet over 21 days. Each point is the mean ± SD (6 determinations). Significance: (a) 1vs 2: p<0.005; 1vs 3: p<0.005; 1vs 4: p<0.001; 2 vs 3: p<0.05; 2 vs 4: p<0.05; 3vs 4: p ns. (b) 1vs 2: p<0.0001; 1vs 3: p<0.005; 1vs 4: p<0.0005; 2 vs 3: p<0.01; 2 vs 4: p<0.05; 3 vs 4: p ns. (TIF) [file pone.0044650.s001.tif]

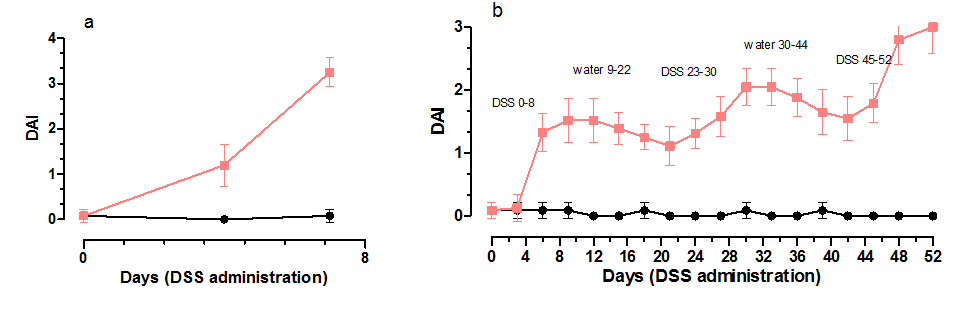

Supplement: Figure S2 — The Disease Activity Index in the acute (a) and chronic colitis (b) model. The DAIs were calculated as described in the Materials and Method section. Each point is the mean ± SD (6 determinations). Where error bars are not shown, these are covered by the point itself. Significance: (a) From day 4 on: DSS vs control tap water: p<0.0001. (b) From day 6 on: DSS vs control tap water: p<0.0001. (TIF) [file pone.0044650.s002.tif]

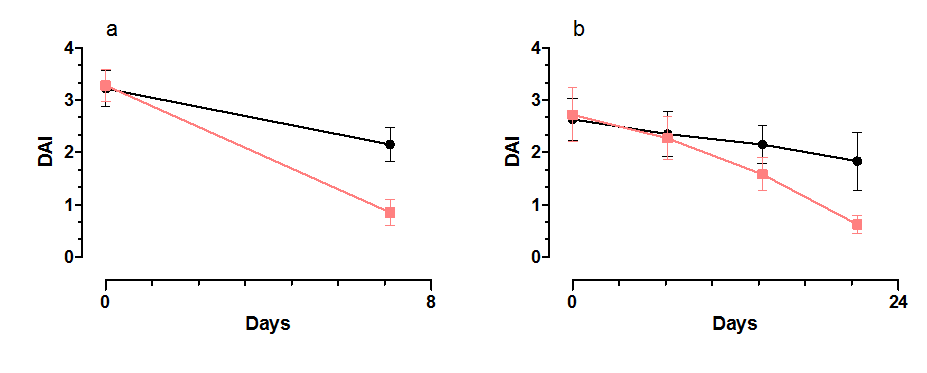

Supplement: Figure S3 — The DAI in mice fed curcuma (red) or standard diet (black) after acute (a) and chronic (b) colitis induction. The DAIs were calculated as described in the Materials and Method section. Each point is the mean ± SD (6 determinations). Significance: (a) 7 days after stopping DSS, Curcuma extract vs control diet, p<0.0001. (b) 14 days after stopping DSS, Curcuma extract vs standard diet: p<0.05; 21 days: p<0.005. (TIF) [file pone.0044650.s003.tif]
