# Supplementary material for: Curcuma longa Extract Exerts a Myorelaxant Effect on the Ileum and Colon in a Mouse Experimental Colitis Model, Independent of the Anti-Inflammatory Effect
Source: PLoS One. 2012 Sep 12;7(9):e44650. doi: 10.1371/journal.pone.0044650 (PMC3440350; doi:10.1371/journal.pone.0044650)
Supplement: Table S1 — Parameters investigated for the evaluation of the Disease Activity Index. (DOC) [file pone.0044650.s004.doc]

**Table S1.** Parameters investigated for the evaluation of the .Disease Activity Index.

| **Score** | **Weight Loss** | **Stool Consistency** | **Blood in Stool** |
| --- | --- | --- | --- |
| **0** | No | Normal | No |
| **1** | 1-5% | Normal | + |
| **2** | 6-10% | Very soft but formed | ++ |
| **3** | 11-15% | Liquid | +++ |
| **4** | >15% | Liquid | Gross rectal bleeding |
