# Supplementary material for: Curcuma longa Extract Exerts a Myorelaxant Effect on the Ileum and Colon in a Mouse Experimental Colitis Model, Independent of the Anti-Inflammatory Effect
Source: PLoS One. 2012 Sep 12;7(9):e44650. doi: 10.1371/journal.pone.0044650 (PMC3440350; doi:10.1371/journal.pone.0044650)
Supplement: Table S2 — Antagonist affinities, expressed as IC50 Values, in the different mouse gut smooth muscle segments. (DOC) [file pone.0044650.s005.doc]

**Table S2.** Antagonist affinities, expressed as IC_50_ Values, in the different mouse gut smooth muscle segments.

| **Mouse Gut Smooth**  **Muscle Segments** | **Curcuma** | |
| --- | --- | --- |
|  | **IC_50_***^a^* | **95% conf lim** |
| Ileum | 0.03 | 0.021–0.044 |
| Distal colon | 0.04 | 0.031–0.042 |

*^a^* IC_50_ was expressed as mg/ml conc. and calculated from concentration-response curves (Probit analysis by Litchfield and Wilcoxon with *n* = 3–5). [RS4].
