# Supplementary material for: Curcuma longa Extract Exerts a Myorelaxant Effect on the Ileum and Colon in a Mouse Experimental Colitis Model, Independent of the Anti-Inflammatory Effect
Source: PLoS One. 2012 Sep 12;7(9):e44650. doi: 10.1371/journal.pone.0044650 (PMC3440350; doi:10.1371/journal.pone.0044650)
Supplement: Table S3 — Potency of Chrabacol and Atropine on isolated mice tissues. (DOC) [file pone.0044650.s006.doc]

**Table S3.** Potency of Chrabacol and Atropine on isolated mice tissues.

| **ileum** | **CCh** | **pEC_50_*^a^*** | 6.63 ± 0.04 |
| --- | --- | --- | --- |
|  | **Atropine** | **p*A*_2_*^b^*** | 8.89 ± 0.03 |
| **colon** | **CCh** | **pEC_50_*^a^*** | 6.08 ± 0.01 |
|  | **Atropine** | **p*A*_2_^b^** | 8.89 ± 0.01 |

*^a^* pEC_50_ = –log EC_50_. EC_50_ values are the means ± SE of at least four independent experiments and were calculated by a non linear regression curve-fitting computer program [SR5]. *^b^*p*A*_2_ values ± SE were calculated from Schild plots [SR6] constrained to slope –1.0 [SR5]. p*A*_2_ is the positive value of the intercept of the line derived by plotting log (DR – 1) vs log [antagonist]. The log (DR – 1) was calculated from three different antagonist concentrations, and each concentration was tested from four to six times. Dose-ratio (DR) values represent the ratio of the potency of the agonist carbachol (EC_50_) in the presence of the antagonist and in its absence. Parallelism of concentration–response curves was checked by linear regression, and slopes were tested for significance (p < 0.05).
