# Supplementary material for: Curcuma longa Extract Exerts a Myorelaxant Effect on the Ileum and Colon in a Mouse Experimental Colitis Model, Independent of the Anti-Inflammatory Effect
Source: PLoS One. 2012 Sep 12;7(9):e44650. doi: 10.1371/journal.pone.0044650 (PMC3440350; doi:10.1371/journal.pone.0044650)
Supplement: Table S4 — Agonist (Carbachol) and antagonist (Atropine) affinities expressed as pEC50 or p A 2 respectively in the isolated mice ileum and distal colon. (DOC) [file pone.0044650.s007.doc]

**Table S4.** Agonist (Carbachol) and antagonist (Atropine) affinities expressed as pEC_50_ or p*A*_2_ respectively in the isolated mice ileum and distal colon.

|  |  |  | **Control** | **Acute Colitis** | | |
| --- | --- | --- | --- | --- | --- | --- |
|  |  |  |  | **After colitis induction** | **Standard Diet**  **after stopping DSS** | **Curcuma extract**  **after stopping DSS** |
| **ileum** | **CCh** | **pEC_50_*^a^*** | 6.63 ± 0.04 | 6.05 ± 0.02 | 5.65 ± 0.05 | 5.61 ± 0.02 |
|  | **Atropine** | **p*A*_2_*^b^*** | 8.89 ± 0.03 | 8.62 ± 0.03 | 8.38 ± 0.01 | 8.47 ± 0.03 |
| **colon** | **CCh** | **pEC_50_*^a^*** | 6.08 ± 0.01 | 5.88 ± 0.04 | 5.65 ± 0.06 | 5.93 ± 0.04 |
|  | **Atropine** | **p*A*_2_^b^** | 8.89 ± 0.01 | 8.84 ± 0.02 | 8.37 ± 0.02 | 8.45 ± 0.04 |

*^a^* pEC_50_ = –log EC_50_. EC_50_ values are the means ± SE of at least four independent experiments and were calculated by a non linear regression curve-fitting computer program [SR5)]. *^b^* p*A*_2_ values ± SE were calculated from Schild plots [SR6] constrained to slope –1.0[RS5] p*A*_2_ is the positive value of the intercept of the line derived by plotting log (DR – 1) *vs* log [antagonist]. The log (DR – 1) was calculated from three different antagonist concentrations, and each concentration was tested from four to six times. Dose-ratio (DR) values represent the ratio of the potency of the agonist carbachol (EC_50_) in the presence of the antagonist and in its absence. Parallelism of concentration–response curves was checked by linear regression, and slopes were tested for significance (p < 0.05). Acute colitis was induced with 5%(w/v) DSS in the drinking water for seven days, and after the period, either curcuma extract or control diet was administered over 7 days.
