# Supplementary material for: Curcuma longa Extract Exerts a Myorelaxant Effect on the Ileum and Colon in a Mouse Experimental Colitis Model, Independent of the Anti-Inflammatory Effect
Source: PLoS One. 2012 Sep 12;7(9):e44650. doi: 10.1371/journal.pone.0044650 (PMC3440350; doi:10.1371/journal.pone.0044650)
Supplement: Table S5 — Agonist (Carbachol) and antagonist (Atropine) affinities expressed as pEC50 or p A 2 respectively in the isolated mice ileum and distal colon. (DOC) [file pone.0044650.s008.doc]

**Table S5.** Agonist (Carbachol) and antagonist (Atropine) affinities expressed as pEC_50_ or p*A*_2_ respectively in the isolated mice ileum and distal colon.

|  |  |  | **Control** | **Chronic Colitis** | | | | | | |
| --- | --- | --- | --- | --- | --- | --- | --- | --- | --- | --- |
|  |  |  |  |  | **7 days** | | **14 days** | | **21 days** | |
|  |  |  |  |  | **Standard Diet** | **Curcuma Extract** | **Standard Diet** | **Curcuma**  **Extract** | **Standard Diet** | **Curcuma**  **Extract** |
| **ileum** | **CCh** | **pEC_50_*^a^*** | 6.63 ± 0.04 | 5.69 ± 0.09 | 5.69 ± 0.03 | 6.79 ± 0.02 | 6.05 ± 0.03 | 6.65 ± 0.07 | 6.02 ± 0.03 | 5.83 ± 0.01 |
|  | **Atrop** | **p*A*_2_*^b^*** | 8.89 ± 0.03 | 9.02 ± 0.02 | 9.09 ± 0.02 | 8.79 ± 0.05 | 8.77 ± 0.02 | 8.77 ± 0.05 | 8.65 ± 0.06 | 8.68 ± 0.04 |
| **colon** | **CCh** | **pEC_50_*^a^*** | 6.08 ± 0.01 | 5.71 ± 0.07 | 5.70 ± 0.01 | 6.37 ± 0.09 | 6.00 ± 0.02 | 6.10 ± 0.09 | 6.01 ± 0.01 | 5.43 ± 0.02 |
|  | **Atrop** | **p*A*_2_*^b^*** | 8.89 ± 0.01 | 9.38 ± 0.01 | 9.44 ± 0.05 | 8.81 ± 0.03 | 8.92 ± 0.01 | 9.16 ± 0.01 | 8.75 ± 0.05 | 8.55 ± 0.04 |

*^a^* pEC_50_ = –log EC_50_. EC_50_ values are the means ± SE of at least four independent experiments and were calculated by a non linear regression curve-fitting computer program[RS5]. *^b^* p*A*_2_ values ± SE were calculated from Schild plot [SR5], constrained to slope –1.0)[SR 4].p*A*_2_ is the positive value of the intercept of the line derived by plotting log (DR – 1) vs log [antagonist]. The log (DR – 1) was calculated from three different antagonist concentrations, and each concentration was tested from four to six times. Dose-ratio (DR) values represent the ratio of the potency of the agonist carbachol (EC_50_) in the presence of the antagonist and in its absence. Parallelism of concentration–response curves was checked by linear regression, and slopes were tested for significance (p < 0.05).
