# Supplementary material for: Curcuma longa Extract Exerts a Myorelaxant Effect on the Ileum and Colon in a Mouse Experimental Colitis Model, Independent of the Anti-Inflammatory Effect
Source: PLoS One. 2012 Sep 12;7(9):e44650. doi: 10.1371/journal.pone.0044650 (PMC3440350; doi:10.1371/journal.pone.0044650)
Supplement: Text S1 — Supporting information. (DOCX) [file pone.0044650.s009.docx]

**Supporting Information**

METHODS

**Details For Acute and Chronic Colitis Induction**

*Acute colitis*: On day 1, mice were weighted and marked in their tail. DSS (5% w/v) was added to the drinking water: The DSS amount consumed was measured daily: 5 ml DSS solution per mouse per day was considered the average amount that a mouse must drink to introduce a sufficient amount of DSS. Whenever it did not occur, the animal was excluded. Control mice received the same drinking water without DSS. The DSS solution was changed every third day up to the seventh day when it was replaced by normal water. Every third day mice were weighted. Stools were evaluated for consistency and presence of blood.

*Chronic colitis*: On day 1, mice were weighted and marked in their tail. DSS (2.5%w/v) was added to the drinking water: 5 ml DSS solution were considered for each mouse. Control mice received the drinking water without DSS. The DSS solution was changed every third day for a total of 8 days administration, after which normal water was replaced for 14 days. The DSS amount consumed was measured daily: 5 ml DSS solution per mouse per day was considered the average amount that a mouse must drink to introduce a sufficient amount of DSS. Whenever it did not occur, the animal were excluded Control mice received the same drinking water without DSS. A similar second and third administration of DSS were after repeated ,with an interval of 14 days. Every third day mice were weighted throughout the experiment. Stools were evaluated for consistency and blood. This administration protocol induces long lasting colonic inflammation in mice [SR1].

**Calculation of the human equivalent dose for mouse of curcumin.**

The calculation of the human equivalent amount of curcuma consumed by mice has been based on the following assumptions: the recommended dose for human is 1.2 g/day and the average adult human weighs 65 kg. This accounts for a dose of 18.4 mg/kg for man. Our calculation of the human equivalent amount of curcuma consumed by mice uses the body surface area normalization method (translational dose) with the following assumptions: a mouse eats 5g chow daily and weighs 22-25 g; chow contains 1g/kg of curcuma. Therefore, 1g / 1000g chow per 5 g chow/day gives 0.005 g daily. If a mouse weighs an average 25 g, then 5 mg/25 g per1000 gives 200 mg/kg daily. As discussed by Reagan-Shaw [SR2], the human equivalent dose (mg/kg) is equal to animal dose (mg/kg) per(animal Km/human Km). As such, human equivalent dose (mg/kg) for mouse is 200mg/kg per (3/37) equal to 16.2 mg/kg, that is consistent with the recommended dose for human of 18.4 mg/kg/day.

**Disease Activity Index Details**

*Evaluation of disease activity index (DAI)*: Body weight, stool consistency and stool blood were recorded every three days. DAI was determined by combining scores of body weight loss, stool consistency and stool blood, divided by 3. Body weight loss was calculated as the percent difference between the initial weight (day 0) and the body weight at each time interval. Blood in stools was determined with the occult blood test kit(Shionogi & Co. Ltd, Osaka, Japan).

The colon was individuated as the segment between the ileo-cecal valve and the rectum and the length was measured. Disease Activity Index (DAI) was calculated for each animal as described by Fitzpatrick [SR3] (see **Table S1**).

RESULTS

*Induction of acute and chronic colitis.* Both in the usual commercial diet and in the curcuma diet animals, the food daily intake was similar. At the end of the induction period, the animals not showing a loss of at least 10% of the initial weight were discharged. A mortality rate of 4% and 2% was observed respectively in acute and chronic colitis groups. At the autopsy, all the deceased animals showed marked distention of the abdomen, in two cases, there was also distension and perforation of the colon with bloody content and blood infiltration of the peritoneum. Appearance of fresh emitted stools varied from frank diarrhea, with traces of blood in acute colitis to amorphous moist pellets with inconstant traces of blood in chronic cases. When a mice didn’t show abnormal feces for at least 7 days before the sacrifice in the chronic and 3 days in the acute experiments respectively, it was not considered suitable for sacrifice. One animal in the acute colitis group and 4 animals in the chronic colitis group were in this way excluded.

*Assessment of acute and chronic colitis and evaluation of the response to curcuma extract.* Length of the colon: in control mice, the colon length was 7.82 ± 0.3 cm; after acute colitis, the colon length was reduced to 5.7 ± 0.5 cm. After curcuma extract over 7 days, it was 6.4 ± 0.6 cm *vs* 5.8 ± 0.5 cm. After chronic colitis similarly, induction it was 4.9 ± 0.5 cm. After 21 days treatment with curcuma, it was 6.3 ± 0.5 *vs* 5.8 ± 0.5 cm of mice without treatment. This shows that shortening of the colon can be reversed by curcuma extract (see **Figure S1**).

Disease Activity Index: DAI during induction of acute and chronic colitis is reported in **Figure S2**: The DAI, which monitors disease severity, increased in mice consuming DSS until the end of the administration. In acute experiments, DAI was already increased at the third day (first determination) of DSS administration. In chronic experiments, DAI was not different in control and DSS treated animals until the sixth day, when there was an exponential increase in DAI that continued up to day 9 in DSS treated mice, when DSS was substituted for with water.

Each following DSS administration was associated with a greater severity of the disease. After induction of acute colitis, DAI slightly decreased over seven days .However, only mice consuming curcuma extract had a significantly lower DAI. Chronic administration of DSS, which has been demonstrated to induce a lasting disease [SR3], is associated with a slow clinical recovery, which is significantly higher in mice consuming curcuma extract (p < 0.005). Significance continued until the end of the experiment (see **Figure S3**).

**Supplementary References (SR)**

SR1. Wirtz S, Neufert C, Weigmann B, Neurath MF. (2007) Chemically induced mouse models of intestinal inflammation. Nat Prot 2: 541-546.

SR2 Reagan-Shaw S, Nihal M, Ahmad N. (2008) Dose translation from animal to human studies revisited. FASEB J 22: 659-61.

SR3 Fitzpatrick LR, Wang J, Le M. (2004) In vitro and in vivo effects of gliotoxin, a fungal metabolite: Efficacy against dextran sodium sulfate-induced colitis in rats. Dig Dis Sci 45: 2327–36.

SR4 Tallarida R J., Murray RB. Manual of Pharmacologic Calculations with Computer Programs, 2nd ed.; Springer-Verlag: New York; 1987.

SRS5 Motulsky H, Christopoulos A. Fitting Models to Biological Data Using Linear and Non Linear Regression, GrafhPad, La Jolla, CA, 2003, www. graphpad.com/manual/Prism4/RegressionBook.pdf. Accessed May 10, 2010.

SRS6 Arunlakshana O, Schild HO. (1997) Some Quantitative Uses of Drug Antagonists 1958. Br J Pharmacol : 120(4 Suppl): 151-61.
